# Has Vietnam Health care funds for the poor policy favored the elderly poor?

**DOI:** 10.1186/1472-6963-12-333

**Published:** 2012-09-22

**Authors:** Nguyen Xuan Thanh, Lars Lindholm

**Affiliations:** 1 Institute of Health Economics, #1200 10405 Jasper Ave, Edmonton, Alberta, T5J 3N4, Canada; 2 Umeå International School of Public Health, Umeå, SE 901 85, Sweden

**Keywords:** Elderly, Health care funds for the poor, Vietnam

## Abstract

**Background:**

The elderly population is increasing in Vietnam. Access to health services for the elderly is often limited, especially for those in rural areas. User fees at public health care facilities and out-of-pocket payments for health care services are major barriers to access. With the aim of helping the poor access public health care services and reduce health care expenditures (HCE), the Health Care Funds for the Poor policy (HCFP) was implemented in 2002. The aim of this study is to investigate the impacts of this policy on elderly households.

**Methods:**

Elderly households were defined as households which have at least one person aged 60 years or older. The impacts of HCFP on elderly household HCE as a percentage of total expenditure and health care utilization were assessed by a double-difference propensity score matching method using panel data of 3,957 elderly households in 2001, 2003, 2005 and 2007, of which 509 were classifies as “treated” (i.e. covered by the policy). Variables included in a logistic regression for estimating the propensity scores to match the treated with the control households, were household and household-head characteristics.

**Results:**

In the first time period (2001–2003) there were no significant differences between treated and controls. This can be explained by the delay in implementing the policy by the local governments. In the second (2001–2005) and third period (2001–2007) the utilizations of Communal Health Stations (CHS) and go-to-pharmacies were significant. The treated were using CHS and pharmacies more between 2001 and 2007 while control households decreased their use.

**Conclusion:**

The main findings suggest HCFP met some goals but not all in the group of households having at least one elderly member. Utilization of CHS and pharmacies increased while the change in HCE as a proportion of total expenditures was not significant. To some extent, private health care and self-treatment are replaced by more utilization of CHS, indicating the poor elderly are better off. However, further efforts are needed to help them access higher levels of public health care (e.g. district health centers and provincial/central hospitals) and to reduce their HCE.

## Background

In 2009 the Vietnamese population reached 86 million, with 70% residing in rural areas [1]. A clear aging trend has been observed during recent decades [2] and the number and proportion of elderly people are expected to grow rapidly during the coming decades. The proportion of people 60 and older was 9.2% in 2006, and this group is projected to increase to 13.4% in 2025 and 26.1% in 2050 [3].

In general, the elderly either live alone, together with a spouse in the same age or in the extended family with all exposed to a double burden of age dependent illness and an unfavorable dependency ratio. Health in the elderly in terms of remaining life expectancy and quality adjusted life years has improved overall but decreased amongst the most vulnerable groups, such as the oldest, the poorest, illiterate and other disadvantaged groups [4,5]. Access to health services by older people is often limited [1], especially for those in rural areas [6]. An unmet need of support for daily care among the elderly has been observed [7] with elderly and their households willing to use and pay for community-based care services [8]. But there is still a lack of social and health networks and resources for long-term elderly care [7].

Elderly care has become one of the prioritized health issues in Vietnam, yet, there is also a lack of both evidence-informed policies for guiding and supporting elderly care, as well as necessary evidence for formulating the policies. One reason may be that elderly people’s living conditions and health has not received much attention in international public health research, despite the fact that they are among the most vulnerable groups in low income countries.

### Health care funds for the poor (HCFP) policy in Vietnam

According to the World Health Report 2005, the Vietnamese budget for public health care, including the central, provincial and communal budgets, was US$ 7 per capita per year in 2002 (about 1.5% of GDP) [9,10]. Out-of-pocket payments accounted for 87.6% of private expenditure on health, which amounts to US$ 16 per capita per year in 2002. The out-of-pocket payments include formal and informal user fees for public services, payments for professional private services, self-medication and pharmaceuticals. The introduction of formal user fees has generated additional income for the public health sector (US$ 0.40 per capita per year in 2001 equal to 7%) [11]. Fees are therefore likely to have become a financial burden on some poor and near-poor households.

To overcome the major barrier that user fees pose to the uninsured, and especially to facilitate access to public hospitals for the uninsured poor, the Prime Minister of Vietnam issued Decision 139 (on October 15, 2002), which established HCFP in each province [12-14]. These funds allocate VND 70,000 (US$ 4.7) per beneficiary per year approximately. Seventy five percent of this fund is covered by the central budget, with the rest covered by other sources, such as individual and community contributions. Provinces can allocate HCFP resources to the direct reimbursement of health care costs, or to the purchase of health insurance cards. By 2003, there were 11 million HCFP beneficiaries (~ 14% of Vietnamese population), representing 84% of the target population [15]. Out of this group, one third had been granted health insurance cards and two thirds had been entitled to direct reimbursements of health care costs. The process of selection of beneficiaries firstly involves identification of those eligible at the village, hamlet and commune level. Lists of eligible households are consolidated and sent to the provincial-level for final selection.

Positive impacts of HCFP policy on general households' health care utilization and expenditure have been published [16-18]. However, little known about what impacts the policy has on vulnerable groups of people, such as the elderly. Thus, the aim of this paper is to investigate the consequences of HCFP for the elderly households.

## Materials

This study was carried out in Bavi, a rural district of Hanoi, about 60 kilometers from the downtown. About 70% of the elderly in Bavi are women and more than 40% of them are widowed [4].

We analyzed a panel data from four re-census surveys (2001, 2003, 2005 and 2007) and four follow-up surveys (each was from January to March in the years of re-census) of FilaBavi to assessed both the short and the long term impacts of the HCFP. FilaBavi is a longitudinal demographic surveillance site covering about 12,000 households with 51,024 people, which accounted for 20% of the population in Bavi district [19]. FilaBavi used a cluster sampling method, in which 67 clusters were randomly selected from a total of 352 clusters in the district. In general, each cluster is a single village. The re-census surveys were performed every second year, gathering information on socio-economic characteristics of households, including housing conditions, water resources, latrines, health care expenditure, total expenditure, total income, agricultural land, access to the nearest commune health centre and hospital, and household socio-economic status (SES) as classified by the local leaders. The follow-up surveys were performed quarterly gathering information on demographic (e.g. age, sex, ethnicity, religion, occupation, education, marital status), morbidity (e.g. number of sick episodes) and health care utilization (e.g. number of use of different types of health care providers) of household members in the last three months. A detail of the FilaBavi longitudinal demographic surveillance site has been published elsewhere [19].

## Methods

We included elderly households (an elderly household was defined as having at least one member aged 60 years or older) and analyzed data at a household level using a double-difference propensity matching method [20-22]. Double difference or difference in difference means the difference between the treated and the untreated in terms of the difference (or change) in the outcomes over time from before to after the treatment. Based on the criteria and the process of selection of beneficiaries regulated in Decision 139, we assumed that all households that the communal people committee classified as “very poor” or “poor” were covered. Furthermore, all households that lived in remote and mountainous areas that were included in Decision 135 [23], were assumed to be covered when the age condition was satisfied. These households are “treated” in terms of propensity score matching (a more general term would be “exposed”). As controls, we tried to find “untreated” (not exposed) households as similar as possible to the treated ones. This was done by matching the propensity scores. The propensity score measures the similarity between “treated” and “untreated” in terms of a vector of observable characteristics. This score is simply the probability of a household being covered by the HCFP. We assumed that there were households that in principle would be eligible for HCFP but not classified as "very poor" or "poor" by the communal people committee, or not included in Decision 135. The two groups should be as similar as possible in pre-treatment characteristics, implying that differences in outcomes can be attributed to the treatment. To estimate the propensity scores we used a logistic regression.

“Treated” and “untreated” cases were matched according to their propensity scores. We selected the nearest (in terms of the propensity score) “untreated” neighbors to a “treated” case. We finally compared the difference in change during the studied periods between “treated” and “untreated” households for the chosen outcome variables. The 95% confidence intervals were estimated by a bootstrap method. We used a balance test to estimate the bias before and after matching.

The chosen outcome variables were the HCE as a percentage of total expenditure, the numbers of use of public health care facilities, including communal health station (CHS), district health center (DHC) and provincial/central hospitals (these were analyzed separately), the number of use of private health care, including private practitioners of western and/or traditional medicine, the number of self-treatment, and the number of go-to-pharmacies to buy drugs with or without advices of the pharmacists or drug sellers.

Variables for estimating the propensity scores to match the treated with the control households were household-head characteristics, including sex, age, religion, ethnicity, marital status, education, and occupation; and household characteristics, including income, expenditure, debt amount, distance to CHS (km), distance to DHC (km), number of members, number of females, number of children under 6 years old, number of people 60 years or older, number of sick episodes, number of children under 6 years old who were sick, number of people 60 years or older who were sick, number of males who were sick, and number of females who were sick. These variables were selected based on potential association between them and the probability of being treated, and also on their availability in the dataset.

Stata software version 9.2 (StataCorp, College Station, Texas, USA) was used for analyses.

These studies was carried out within the FilaBavi in Vietnam, a collaborative research project between Vietnam and Sweden, and got permission from Ministry of Health of Vietnam, local authorities and inform consent from inhabitants. Ethical approval was also given by the Research Ethics Committee at Umeå University, Sweden.

## Results

In total, 3957 elderly households were included and 509 were classifies as “treated”, i.e. covered by this reform. In Table 1 descriptive statistics are presented. The overall pattern is that health care expenditures as part of total expenditures dropped in both groups, as well as the total number of utilizations. Within this pattern there was an increase in the use of public services and a decrease in the use of private, and a pronounced decrease of self-treatment. Go-to-pharmacy was the most common services during the whole period.

**Table 1:** Descriptive statistics - outcome variables by years

**Outcome variables**	**Treated (N = 509)**		**Control (N = 3448)**	
	**Mean**	**SD**	**Mean**	**SD**
**2001**				
HCE as % of total expenditure (%)	8.8%	13.5%	7.7%	12.4%
Number of CHS utilizations (#)	0.075	0.298	0.093	0.430
Number of DHC utilizations (#)	0.096	0.333	0.092	0.328
Number of provincial/central hospital utilizations (#)	0.033	0.201	0.034	0.200
Number of private health care utilizations (#)	0.876	1.097	0.896	1.237
Number of self-treatment (#)	1.012	1.260	1.015	1.318
Number of go-to-pharmacy (#)	1.741	1.390	1.744	1.513
Number of other types of health care (#)	0.008	0.108	0.005	0.076
Total number of utilizations (#)	3.841	3.170	3.879	3.476
**2003**				
HCE as % of total expenditure (%)	6.1%	9.9%	6.5%	12.0%
Number of CHS utilizations (#)	0.096	0.412	0.098	0.441
Number of DHC utilizations (#)	0.079	0.323	0.082	0.295
Number of provincial/central hospital utilizations (#)	0.035	0.195	0.032	0.184
Number of private health care utilizations (#)	0.796	1.084	0.844	1.159
Number of self-treatment (#)	0.674	0.918	0.712	1.024
Number of go-to-pharmacy (#)	1.371	1.204	1.426	1.319
Number of other types of health care (#)	0.006	0.077	0.007	0.095
Total number of utilizations (#)	3.057	2.758	3.201	3.056
**2005**				
HCE as % of total expenditure (%)	6.1%	11.1%	6.5%	12.8%
Number of CHS utilizations (#)	0.183	0.540	0.106	0.387
Number of DHC utilizations (#)	0.126	0.382	0.088	0.331
Number of provincial/central hospital utilizations (#)	0.024	0.176	0.031	0.182
Number of private health care utilizations (#)	0.434	0.805	0.497	0.859
Number of self-treatment (#)	0.548	0.830	0.473	0.747
Number of go-to-pharmacy (#)	1.652	1.697	1.461	1.535
Number of other types of health care (#)	0.004	0.089	0.003	0.068
Total number of utilizations (#)	2.971	2.570	2.660	2.455
**2007**				
HCE as % of total expenditure (%)	6.5%	12.8%	6.3%	11.9%
Number of CHS utilizations (#)	0.169	0.473	0.104	0.412
Number of DHC utilizations (#)	0.136	0.411	0.134	0.426
Number of provincial/central hospital utilizations (#)	0.029	0.169	0.036	0.195
Number of private health care utilizations (#)	0.448	0.796	0.514	0.869
Number of self-treatment (#)	0.564	0.884	0.496	0.829
Number of go-to-pharmacy (#)	1.717	1.834	1.620	1.730
Number of other types of health care (#)	0.004	0.063	0.002	0.048
Total number of utilizations (#)	3.067	2.850	2.906	2.728

HCE = Health care expenditure; CHS = Communal health station; DHC = District health center.

Table 2 presents the results from the logistic model used for estimating the propensity scores. The probability that a household are “treated” depended significantly on the age, education and occupation of the household head, the total income, debt, the number of females, and the number of people aged 60 or more of the household.

**Table 2:** Logistic regressions to estimate propensity score

**Dependent variable: Treated households**				
**Independent variables:**	**Coef.**	**95% CI**		**Sig.**
***1. Household head characteristics***				
Age of household head in years	−0.016	−0.026	−0.005	**yes**
Sex of household head-Male	−0.255	−0.598	0.089	no
Number of years in school of household head	−0.089	−0.135	−0.043	**yes**
Ethnic of household head-Kinh	−0.337	−0.842	0.168	no
Religion of household head-Non religion	−0.369	−0.955	0.218	no
Marital status of household head-Divorced	0.161	−0.494	0.815	no
Marital status of household head-Widowed	−0.086	−0.450	0.277	no
Marital status of household head-Single	0.100	−0.540	0.740	no
Occupation of household head-Retire	−1.090	−1.714	−0.467	**yes**
Occupation of household head-Elderly	−0.031	−0.344	0.283	no
Occupation of household head-Other	0.216	−0.072	0.504	no
***2. Household characteristics***				
Distance to commune health station (km)	−0.096	−0.209	0.016	no
Distance to district health center (km)	−0.023	−0.047	0.001	no
Total income last year	0.000	0.000	0.000	**yes**
Total expenditure last year	0.000	0.000	0.000	no
Debt amount	0.000	0.000	0.000	**yes**
Number of females in household	0.118	0.009	0.226	**yes**
Number of children under 6 years in household	0.175	−0.037	0.387	no
Number of people > =60 years in household	−0.270	−0.497	−0.043	**yes**
Number of children under 6 years who were sick	−0.120	−0.414	0.173	no
Number of people > =60 who were sick	0.059	−0.169	0.287	no
Number of males who sick	0.113	−0.038	0.265	no
Number of females who were sick	0.029	−0.120	0.178	no
**Constant**	2.089	0.970	3.208	**yes**

N = 3957; LR chi2(23) = 557.58; Prob > chi2 = 0.000; Log likelihood = −1239.82.

In Table 3 the outcome variables are shown. In the first time period (2001–2003) there were no significant differences between treated and controls. This result can be explained by the delay in implementing the policy by the local governments.

**Table 3:** Impacts of the HCFP on household HCE and health care utilization

	**Change between years**					
	**Treated**	**Control**	**Difference in changes between treated and control**			
**2003-2001**			**mean**	**95%CI**		**Sig.**
HCE as % of total expenditure (%)	−2.8%	−1.7%	−1.2%	−3.3%	0.7%	no
Number of CHS utilizations (#)	0.017	−0.045	0.062	0.000	0.152	no
Number of DHC utilizations (#)	−0.015	−0.013	−0.002	−0.044	0.065	no
Number of provincial/central hospital utilizations (#)	0.004	0.000	0.004	−0.036	0.045	no
Number of private health care utilizations (#)	−0.058	−0.011	−0.047	−0.278	0.118	no
Number of self-treatment (#)	−0.357	−0.286	−0.071	−0.325	0.124	no
Number of go-to-pharmacy (#)	−0.365	−0.297	−0.068	−0.302	0.241	no
Number of other types of health care (#)	0.006	−0.006	0.013	0.000	0.026	no
Total number of utilizations (#)	−0.767	−0.658	−0.109	−0.703	0.414	no
**2005-2001**						
HCE as % of total expenditure (%)	−2.4%	−1.5%	−1.0%	−4.5%	1.1%	no
Number of CHS utilizations (#)	0.118	−0.079	0.197	0.144	0.272	**yes**
Number of DHC utilizations (#)	0.032	0.013	0.019	−0.023	0.070	no
Number of provincial/central hospital utilizations (#)	−0.006	−0.015	0.009	−0.022	0.056	no
Number of private health care utilizations (#)	−0.410	−0.521	0.111	−0.014	0.243	no
Number of self-treatment (#)	−0.487	−0.519	0.032	−0.171	0.182	no
Number of go-to-pharmacy (#)	−0.053	−0.393	0.340	0.108	0.753	**yes**
Number of other types of health care (#)	0.004	−0.009	0.013	0.002	0.024	**yes**
Total number of utilizations (#)	−0.803	−1.524	0.720	−0.121	1.197	no
**2007-2001**						
HCE as % of total expenditure (%)	−2.0%	−2.4%	0.3%	−1.6%	3.6%	no
Number of CHS utilizations (#)	0.096	−0.058	0.154	0.073	0.229	**yes**
Number of DHC utilizations (#)	0.043	0.047	−0.004	−0.056	0.045	no
Number of provincial/central hospital utilizations (#)	0.000	0.004	−0.004	−0.032	0.031	no
Number of private health care utilizations (#)	−0.382	−0.502	0.120	−0.094	0.348	no
Number of self-treatment (#)	−0.457	−0.402	−0.056	−0.338	0.100	no
Number of go-to-pharmacy (#)	0.028	−0.248	0.276	0.034	0.641	**yes**
Number of other types of health care (#)	0.004	−0.006	0.011	−0.005	0.018	no
Total number of utilizations (#)	−0.669	−1.165	0.496	−0.064	1.051	no

HCFP = Health care funds for the poor; HCE = Health care expenditure; CHS = Communal health station; DHC = District health center.

In the second period (2001–2005) and the third period (2001–2007) the utilizations of CHS and go-to-pharmacy were significant. The treated households utilized CHS and pharmacies more between 2001 and 2007, while control households decreased their use.

## Discussion

The main findings in this study suggest the HCFP policy met some goals, but not all in the group of households which have at least one elderly member. Utilization of CHS and pharmacies increased, while the change in health care expenditures as a proportion of total expenditures was similar between the treated and controls. The decrease in the relative size of health care expenditures can be explained by the income increase during this period – about 300% in running prices. Summarizing this time period, the conclusion is that the treated are better off. To some extent, private care and self-treatment are replaced by more utilization of CHS and pharmacies.

Private health care in Vietnam is a very heterogeneous entity, covering both specialized hospital treatment at international standards, as well as local healers without any formal medical education. The volume of contacts in Bavi are retired public health staff who open a clinic, current public health staff who work after-hours, and traditional healers. Because the quality of private health care services is not better than public services [24], the increase of public health utilization by the HCFP really benefits the poor.

The consequences of this policy in the total poor population have been discussed in the literature. For example, Wagstaff [16] found a mixed result: The policy increased the utilization of health care and reduced the risk of catastrophic out-of-pocket spending. On the other hand, the average out-of-pocket spending did not change. Further, the utilization impact was more pronounced for inpatient care than outpatient care, and the impact was larger among the better off. In Axelson et al.’s study [17], the main result was that the policy achieved its objectives of increased public health care utilization and reduction of the out-of-pocket spending in the target population. Both studies focused on a short-term impact of HCFP, and neither included the health care expenditure as a percentage of total expenditure, which could make more sense than the absolute amount of health care expenditure for an over-time comparison, especially in a developing economy with high inflation such as Vietnam. Thanh et al. [18] found the portion of total expenditures used for health care decreased and the use of CHS increased. Thus, the policy got more extensive consequences among the poor in general than in the poor elderly households.

We used the same analytical approach used in Thanh et al. [18], but included only elderly households. The double-difference propensity score matching technique could take care of time-invariant unobservable variables (fixed effects). Our baseline (2001) is clearly before the time point when the reform was implemented (2002). The extent to which bias is reduced by the matching depends on the richness and quality of the control variables (i.e. the independent variables in the logistic regression). We used 23 control variables out of which 7 were significant. The balance test results (Table 4) indicated that the bias dramatically reduced after matching.

**Table 4:** Pseudo R2 and absolute standardized bias before and after matching

	**Before matching**	**After matching**
Pseudo R2	0.178	0.011
Mean bias	19.559	3.904
Median Bias	12.766	3.205
SD of bias	18.999	3.367
Minimum bias	3.104	0.295
Maximum bias	61.454	14.209
Number of Explanatory variables	23	23

A potential problem in this kind of study is that many variables regard the household, not the elderly persons as individuals. This is natural and logical in some cases, such as economic resources in the household and health care expenditures as a portion of total expenditures or income; yet in other cases, such as utilization of care, individual data would be more suitable. We only know the utilization per household, and have to assume the same pattern in treated and control households, i.e. that the elderly members get the same share in both types when utilization change. Considering the methods adopted, and our understanding of the cultural context, this assumption seems to be reasonable.

## Conclusion

In conclusion, the results indicate that HCFP met some goals but not all in the group of households having at least one elderly member. Utilization of CHS and pharmacies increased while the change in HCE as a proportion of total expenditures was not significant. To some extent, private health care and self-treatment are replaced by more utilization of CHS, indicating the poor elderly are better off; however, further efforts are needed to help them access higher levels of public health care (e.g. DHC and provincial/central hospitals) and to reduce their HCE as a proportion of total expenditures.

## Competing interests

The authors declare that they have no competing interests.

## Authors' contributions

NXT designed the study, analyzed and interpreted the data, participated in drafting, and revised the manuscript. LL contributed to conception and design of study, acquisition and interpretation of data, and drafted the manuscript. Both authors read and approved the final manuscript.

## Pre-publication history

The pre-publication history for this paper can be accessed here:

http://www.biomedcentral.com/1472-6963/12/333/prepub
